# Plasma Amyloid Is Associated with White Matter and Subcortical Alterations and Is Modulated by Age and Seasonal Rhythms in Mouse Lemur Primates

**DOI:** 10.3389/fnagi.2018.00035

**Published:** 2018-02-14

**Authors:** Charlotte Gary, Anne-Sophie Hérard, Zoé Hanss, Marc Dhenain

**Affiliations:** ^1^Centre National de la Recherche Scientifique, Université Paris-Sud, Université Paris-Saclay, UMR 9199, Neurodegenerative Diseases Laboratory, Fontenay-aux-Roses, France; ^2^Commissariat à l'Energie Atomique et aux Energies Alternatives, Direction de la Recherche Fondamentale, Institut François Jacob, MIRCen, Fontenay-aux-Roses, France

**Keywords:** Alzheimer, lemur, plasma amyloid, brain morphometry, aging, seasons

## Abstract

Accumulation of amyloid-β (Aβ) peptides in the brain is a critical early event in the pathogenesis of Alzheimer's disease (AD), the most common age-related neurodegenerative disorder. There is increasing interest in measuring levels of plasma Aβ since this could help in diagnosis of brain pathology. However, the value of plasma Aβ in such a diagnosis is still controversial and factors modulating its levels are still poorly understood. The mouse lemur (*Microcebus murinus*) is a primate model of cerebral aging which can also present with amyloid plaques and whose Aβ is highly homologous to humans'. In an attempt to characterize this primate model and to evaluate the potential of plasma Aβ as a biomarker for brain alterations, we measured plasma Aβ_40_ concentration in 21 animals aged from 5 to 9.5 years. We observed an age-related increase in plasma Aβ_40_ levels. We then evaluated the relationships between plasma Aβ_40_ levels and cerebral atrophy in these mouse lemurs. Voxel-based analysis of cerebral MR images (adjusted for the age/sex/brain size of the animals), showed that low Aβ_40_ levels are associated with atrophy of several white matter and subcortical brain regions. These results suggest that low Aβ_40_ levels in middle-aged/old animals are associated with brain deterioration. One special feature of mouse lemurs is that their metabolic and physiological parameters follow seasonal changes strictly controlled by illumination. We evaluated seasonal-related variations of plasma Aβ_40_ levels and found a strong effect, with higher plasma Aβ_40_ concentrations in winter conditions compared to summer. This question of seasonal modulation of Aβ plasma levels should be addressed in clinical studies. We also focused on the amplitude of the difference between plasma Aβ_40_ levels during the two seasons and found that this amplitude increases with age. Possible mechanisms leading to these seasonal changes are discussed.

## Introduction

Amyloid-β (Aβ) is a peptide generated after proteolytic cleavage of its precursor, the amyloid precursor protein (APP), by β- and γ-secretases (Haass, 2004). After their production in the brain, Aβ peptides can aggregate into amyloid plaques that initiate neurotoxic events, neuronal loss, and tau-associated pathology leading to Alzheimer's disease (AD), the most common age-related neurodegenerative disorder. This aggregation of Aβ is thought to result from increased local brain Aβ concentration above a critical threshold (Burgold et al., 2014) because of an overproduction or a reduced clearance of Aβ. This clearance can be related to the degradation of Aβ in the brain (Iwata et al., 2001) and/or to Aβ exportation outside the brain through the blood–brain barrier (BBB) (Shibata et al., 2000) and/or via the interstitial fluid (ISF) bulk flow into the cerebrospinal fluid (CSF), and from there into the bloodstream (Silverberg et al., 2003). Amyloid is also found in peripheral tissues. For example, high Aβ_40_ levels are reported in the aorta in patients with advanced atherosclerotic lesions (Roher et al., 2009) and in the plasma of patients with coronary artery diseases, cardiovascular diseases, or diabetes mellitus (Janelidze et al., 2016; Roeben et al., 2016). During physiological aging, some studies (Lopez et al., 2008; Huang et al., 2013), though not all (Roher et al., 2009), reported increased baseline plasma Aβ levels too. These changes in peripheral Aβ levels can be attributed to either clearance of cerebral Aβ, or to Aβ synthesis at the level of peripheral sources such as skeletal muscle, platelets, and vascular walls which can produce appreciable amounts of Aβ (Roher et al., 2009). Altered peripheral clearance such as that associated with renal function could also increase plasma Aβ levels (Jin et al., 2017).

Despite the presence of Aβ in brain pathology, the relationship between plasma Aβ levels and other pathology markers is still unresolved. Recent longitudinal studies have demonstrated a clear link between plasma Aβ and the risk of dementia in an AD context (Head et al., 2011; Chouraki et al., 2015) or a correlation with AD diagnosis and brain amyloid burden (Rembach et al., 2014), while some others have ruled out the predictive value of plasma Aβ in AD risk determination (Lövheim et al., 2017). Some studies in AD and MCI patients suggested a relationship between high Aβ_42_ and/or Aβ_40_ levels and white matter lesions (Gurol et al., 2006; Janelidze et al., 2016). Studies in dementia-free persons reported mixed findings. Some reported that higher Aβ_42_ and/or Aβ_40_ levels are associated with larger white matter hyperintensities (WMH) and lacunar infarcts (van Dijk et al., 2004; Gurol et al., 2006; Hilal et al., 2017). Patients bearing MRI-defined brain infarcts had higher Aβ_42_ levels (Toledo et al., 2011). In dementia-free persons, high plasma Aβ_40_ was correlated with hippocampal atrophy and with increased rate of cerebral atrophy (Kaffashian et al., 2015). However, in some studies, opposite results were obtained and a reduced plasma Aβ_40_ was associated with increased progression of WMH (Kaffashian et al., 2014).

Animal models are widely used to study cerebral pathologies and aging, to evaluate new drugs and to assess biomarkers. Among various species stands the mouse lemur (*Microcebus murinus*), a small (70–150 g) primate model of cerebral aging. It has a short lifespan of ~12 years in captivity (Languille et al., 2012) and an Aβ peptide that is completely homologous to that of humans (Silhol et al., 1996). Approximately 10% of lemurs over 6 years old develop intracerebral Aβ deposits and their amyloid plaque load is usually low (Mestre-Frances et al., 2000; Kraska et al., 2011), though a previous study reported great heterogeneity of plasma Aβ_40_ concentrations in old animals from low to high (Roy et al., 2015). Low plasma Aβ_40_ levels in old animals have been associated with high cerebral intracellular labeling with antibodies specific of amyloid and APP (Roy et al., 2015). As mouse lemurs can spontaneously develop age-related cerebral atrophy (Kraska et al., 2011; Picq et al., 2012; Sawiak et al., 2014), they are widely used to evaluate parameters modulating cerebral atrophy (Djelti et al., 2016).

One of the biological features of mouse lemurs is that their metabolic and physiological parameters undergo seasonal changes that are strictly controlled by photoperiodic variations (Dal-Pan et al., 2011). Exposure to day lengths shorter than 12 h (i.e., 10 h light/day) results in complete sexual rest, fattening, lethargy, reduced behavioral activities, and torpor. Torpor is defined as a state during which body temperature and metabolic rate are significantly reduced. In contrast to hibernation, torpor is characterized by short-duration bouts that do not exceed 24 h, so that animals exhibit active states each day between torpor episodes (Perret and Aujard, 2001). Exposure to day lengths greater than 12 h (i.e., 14 h light/day) induces sexual activity, increases in behavioral activities, and high hormonal levels (Perret, 1997). Thus, two marked biological seasons called “winter” and “summer” occur in lemurs. They correspond respectively to periods of “lower” or “higher” activity levels.

In the present study, we assessed plasma Aβ_40_ levels in 21 adult mouse lemurs (aged from 5 to 9.5 years-old at the beginning of the study) and confirmed previous studies demonstrating higher plasma Aβ levels in the oldest animals. MR images were recorded for each animal in order to evaluate relationships between plasma Aβ_40_ levels and cerebral pathology. A voxel-based morphometry (VBM) study revealed a relationship between lower plasma Aβ_40_ levels and atrophy of white matter and subcortical brain regions. We also investigated the seasonal rhythmicity of plasma Aβ_40_ in mouse lemurs by measuring its concentration during two consecutive seasons. Mouse lemurs displayed strong seasonal variations of plasma Aβ_40_ levels with an increase in winter. We examined the amplitude of the difference between plasma Aβ_40_ levels in the two seasons and found that this amplitude increased with aging.

## Materials and methods

### Animals and biological rhythms

This study was carried out in accordance with the recommendations of the European Communities Council directive (2010/63/EU). The protocol was approved by the local ethics committees CEtEA-CEA DSV IdF (authorizations 201506051 736524 VI, APAFIS#778). All mouse lemurs were born in a laboratory breeding colony (Brunoy, France, authorization n°E91-114-1), and maintained at steady ambient temperature (24–26°C) and relative humidity (55%). Their seasonal and daily rhythms were controlled by an artificial photoperiod regimen of alternating 6 month seasons of short day lengths (10 h light/day, “winter”) and long day lengths (14 h light/day, “summer”). The shift from one season to the other was made without progressive transition. Animals were fed with fresh fruits, milky mixture (eggs, cereals, milk cheese, and honey bread) and meal worms.

Twenty-one mouse lemurs (5–9.5 years old) were involved in this study. They were split into two groups according to age at first blood sampling: middle-aged (fifteen animals, 5–6 years old) and old (six animals over 7 years old). Two blood samples were collected from each animal: the first one was taken during winter, 1 month after the shift in the season, and the second one was obtained 6 months later during summer. MRI images were recorded for each animal.

### Blood collection, pre-treatment, and plasma Aβ detection

Plasma Aβ_40_ was measured as previously described (Roy et al., 2015). Briefly, blood was sampled at the beginning of the day after an overnight fast. This corresponds to the inactive period for the nocturnal lemurs. In mouse lemurs, blood has to be collected from the saphenous vein. The vein was pricked with a needle at a 45° angle and blood drops were collected in small heparinized hematocrit capillaries (60 μl, Hirschmann-Laborgeraete, ref 91 00 260) in which the blood penetrated by capillarity. All capillaries were kept on ice and centrifuged (× 2,000 g; 10 min) at +4°C immediately after collection. For each animal and at each time point, the plasma layer taken from the centrifuged hematocrit capillaries was then aliquoted into 200 μl polypropylene tubes (Corning, Thermowell® Gold PCR tubes, Product #3745) to avoid freeze-thaw cycles. Volumes ranging from 120 to 200 μl of plasma could be sampled for each animal. A cocktail of protease inhibitors (Complete Mini; Roche, Meylan, France) was added to each plasma sample at a final concentration of 1X. The aliquots were frozen at −80°C within 1 h of sampling and kept frozen until analysis. This preanalytical processing corresponds to previous recommendations (Watt et al., 2012) except for the use of heparin instead of EDTA as anticoagulant. We chose heparin because, to the best of our knowledge, the only anticoagulant-coated hematocrit capillaries commercially available use heparin as the anticoagulant. Using heparin probably resulted in lower Aβ values in our samples as compared to those that would have been obtained with EDTA (Watt et al., 2012), but this has not been reported to change intra-experiment values (Lachno et al., 2009).

As mouse lemurs constitutively produce Aβ peptides, their plasma Aβ concentrations are much lower than those of transgenic mice overexpressing mutated forms of APP, so mouse lemur Aβ_42_ levels are usually below the limit of detection with classical enzyme-linked immunosorbent assay (ELISA) tests (Roy et al., 2015). We thus focused on Aβ_40_ as its high relative concentration compared to other Aβ species allowed measurements above the lower limit of quantification from very small volumes of plasma. It is the main Aβ component in the brain and is strongly correlated to Aβ_42_ in humans (Huang et al., 2013). Plasma Aβ_40_ levels were measured in duplicate using ELISA “Human β amyloid 1–40” kits (Invitrogen, Saint Aubin, France) following the manufacturer's protocol for non-diluted plasma samples. With this kit, 50 μl of plasma is required to perform one measure. Antibodies used in these kits are coated with monoclonal antibodies against the N-terminal part of human Aβ and the secondary antibodies are rabbit antibodies specific for the C-terminal part of the human Aβ_40_ sequence. The calibration standards (synthetic Aβ_40_ peptides) were provided with the kits. The calibration standards and negative controls always performed within the manufacturer's range and samples' Aβ concentrations always stood within the calibration range.

### MRI acquisition and analysis

Cerebral imaging was performed by MRI in all the animals involved in the study. Brain images were recorded on a 7.0 Tesla spectrometer (Agilent, USA) using a four channel phased-array surface coil (Rapid Biomedical, Rimpar, Germany) actively decoupled from the transmitting birdcage probe (Rapid Biomedical, Rimpar, Germany). Briefly, animals were anesthetized by isoflurane (4% for induction and 1–1.5% for maintenance). Respiratory rate was monitored to ensure animal stability until the end of the experiment. Body temperature was maintained by an air heating system. Two-dimensional fast spin echo images were recorded with an isotropic nominal resolution of 230 μm (128 slices, TR/TE = 10,000/17.4 ms; rare factor = 4; field of view = 29.4 × 29.4 mm^2^, matrix = 128 × 128, slice thickness = 230 μm, acquisition time = 32 min).

Images were analyzed by VBM using SPM8 (Wellcome Trust Institute of Neurology, University College London, UK, www.fil.ion.ucl.ac.uk/spm) with the SPMmouse toolbox (http://spmmouse.org) dedicated to animal brain morphometry (Sawiak et al., 2014). VBM is a reference method, widely used to identify structural changes in the brain including in humans (Whitwell, 2009).

Brain images were segmented into three tissue probability maps (tpm) corresponding to tissues with cortical gray matter (GM), white matter and subcortical nuclei (WM-SC), and cerebrospinal fluid (CSF) characteristics, using locally developed priors. The intensity of the pixel in each probability map represents the probability of the pixel to be GM, WM, or CSF. Then brain images and tpm were spatially transformed to the standard space, defined by Sawiak et al., using a GM mouse lemur template (Sawiak et al., 2014). Affine regularization was set for an average-sized template, with a bias non-uniformity FWHM cut off of 10 mm, a 5 mm basis function cut off and a sampling distance of 0.3 mm. The resulting GM and WM-SC portions were output in rigid template space, and DARTEL (Ashburner, 2007) was used to create non-linearly registered maps for each subject and common templates for the cohort of animals. The warped GM and WM-SC portions for each subject were adjusted using the Jacobian determinant from the DARTEL registration fields to preserve tissue amounts (“optimized VBM;” Good et al., 2001) and smoothed with a Gaussian kernel of 600 μm to produce statistical maps (T maps) for analysis.

A first general linear model (GLM) was designed to evaluate relative changes in GM and WM-SC tpm values, a parameter reflecting atrophy, as a function of age. The sex of the animals and total intracranial volumes (TIV) were considered in the design matrix and were treated as covariates of no interest. More specifically, with the GLM, if the brain of one animal is defined by the number “*j*,” and the location of a pixel is defined as “*k*.” The signal within a pixel ($Y_{j}^{k}$) can be explained by the following equation

$$Y_{j}^{k}=\beta_{1}^{k}+x_{j,1}\beta_{2}^{k}+S_{j,1}\beta_{5}^{k}+S_{j,2}\beta_{6}^{k}+TIV_{j}\beta_{7}^{k}+\epsilon_{j}^{k}\;(GLM-1)$$

With $\beta_{1}^{k}$ = Mean image; $\beta_{2}^{k}$ = Evolution of the signal according to the age of the animals (n = 21 animals); $\beta_{5}^{k}$ = Sex effect on signal for males; $\beta_{6}^{k}$ = Sex effect on signal for females; $\beta_{7}^{k}$ = effect of TIV on the signal for each animal. In this matrix, *x*_*j*,1_ corresponds to the age of each animal *j*. *S*_*j*,1_ and *S*_*j*,2_ correspond to the sex of the animals. *S*_*j*,1_ = 1 if the animal *j* is a male and = 0 otherwise; *S*_*j*,2_ = 1 if the animal *j* is a female and = 0 otherwise.

A second GLM was designed to evaluate the relationships between GM and WM-SC tpm values and plasma Aβ_40_ levels. Winter and summer plasma Aβ_40_ levels were added in the model. For this study, age, sex and TIV were considered in the design matrix and were treated as covariates of no interest. More specifically, with this second GLM, the signal within a pixel ($Y_{j}^{k}$) can be explained by the following equation

$$\begin{array}{l} Y_{j}^{k}=\beta_{1}^{k}+x_{j,1}\beta_{2}^{k}+x_{j,2}\beta_{3}^{k}+x_{j,3}\beta_{4}^{k}+S_{j,1}\beta_{5}^{k}+S_{j,2}\beta_{6}^{k}\\ \;+TIV_{j}\beta_{7}^{k}+\epsilon_{j}^{k}\;(GLM-2) \end{array}$$

With $\beta_{1}^{k}$ = Mean image; $\beta_{2}^{k}$ = Evolution of the signal according to the age of the animals (*n* = 21 animals); $\beta_{3}^{k}$ = Evolution of the signal according to winter plasma Aβ_40_ levels; $\beta_{4}^{k}$ = Evolution of the signal according to summer plasma Aβ_40_ levels; $\beta_{5}^{k}$ = Sex effect on signal for males; $\beta_{6}^{k}$ = Sex effect on signal for females; $\beta_{7}^{k}$ = effect of TIV on the signal for each animal. *x*_*j*,1_, *x*_*j*,2_ and *x*_*j*,3_ correspond to the age, winter plasma Aβ levels and summer plasma Aβ levels for each animal *j*. *S*_*j*,1_ and *S*_*j*,2_ correspond to the sex of the animals. *S*_*j*,1_ = 1 if the animal *j* is a male and = 0 otherwise; *S*_*j*,2_ = 1 if the animal *j* is a female and = 0 otherwise.

As an example, on the basis of the GLM-2 model, for the first animal (a 7.8 year-old female, winter and summer plasma Aβ_40_ levels = 75.2 and 35.5 pg/ml with a TIV of 2150 mm^3^):

$$Y_{1}^{k}=\beta_{1}^{k}+7.8\;\beta_{2}^{k}+75.2\;\beta_{3}^{k}+\;35.5\;\beta_{3}^{k}+\beta_{6}^{k}+2150\;\beta_{7}^{k}+\epsilon_{1}^{k};$$

for the second animal (a 5.6 year-old male, winter and summer plasma Aβ levels = 37.3 and 33.5 pg/ml with a TIV of 2058 mm^3^)

$$Y_{2}^{k}=\beta_{1}^{k}+5.6\;\beta_{2}^{k}+37.3\;\beta_{3}^{k}+\;33.5\;\beta_{3}^{k}+\beta_{5}^{k}+2058\;\beta_{7}^{k}+\epsilon_{2}^{k}$$

and so on for the other animals.

A contrast defines a linear combination of β as *c*^*T*^β. For example, the test evaluating the positive relationship between plasma Aβ levels and the probability of pixels being GM is defined using a contrast *c*^*T*^β = {0 0 1 1 0 0 0]^T^. The Null hypothesis is $H_{0}:\;c^{T}\beta =0$, whereas the alternative hypothesis is $H_{1}:\;c^{T}\beta >0$. This hypothesis is tested with:

$$T=\frac{c^{T}\beta }{\sqrt{\sigma^{2}c^{T}{\left(X^{T}X\right)}^{-1}c}}=\frac{\text{contrast}}{\sqrt{\text{estimated variance}}}$$

This analysis allows the removal of confounding effects, such as age ($\beta_{2}^{k}$), sex ($\beta_{5}^{k}$ and $\beta_{6}^{k}$), or TIV ($\beta_{7}^{k}$) from the raw data. In other words, volumetric scans were entered as the dependent variable. Depending on the tested hypothesis, aging or plasma Aβ levels were the independent variables. Aging, sex, and TIV were covariates.

One-tailed *t*-test contrasts were set up to find areas in which probability values from GM or WM-SC maps correlated with age or plasma Aβ levels. To control for multiple comparisons, an adjusted *p*-value was calculated using the voxel-wise false discovery rate (FDR-corrected *p* < 0.05), with extent threshold values of 500 voxels, meaning that clusters required 500 contiguous voxels to be selected as relevant (Genovese et al., 2002). Voxels with a modulated GM value below 0.2 were not considered for statistical analysis. The operator was blinded to the animal names during image processing. This type of regression technique produces t-statistic and color-coded maps that are the product of a regression model performed at every voxel in the brain. Contiguous groups of voxels that attain statistical significance, called clusters, are displayed on brain images.

One of the minor limitations of this study is that brain images were recorded at different time points surrounding the winter and summer blood sampling, so it was not possible to attribute MR images to a given season. However, to our knowledge, modulation of cerebral atrophy by seasonal effects has never been described in mouse lemurs. We thus consider that brain images correspond to the state of an animal the year of the study independently of the season.

### Statistical analysis

Paired Student's *t*-tests were used to evaluate seasonal effects on plasma Aβ_40_ levels. Pearson's tests were used to evaluate the correlation between plasma Aβ_40_ concentrations, or amplitude of seasonal variations of Aβ_40_ and age. Statistical analysis was done using Statistica 7.1 software (StatSoft, Maisons-Alfort, France). *P* < 0.05 was set as the level of statistical significance for each test.

## Results

### Plasma Aβ_40_ is modulated by age and season

**Figure 1:**
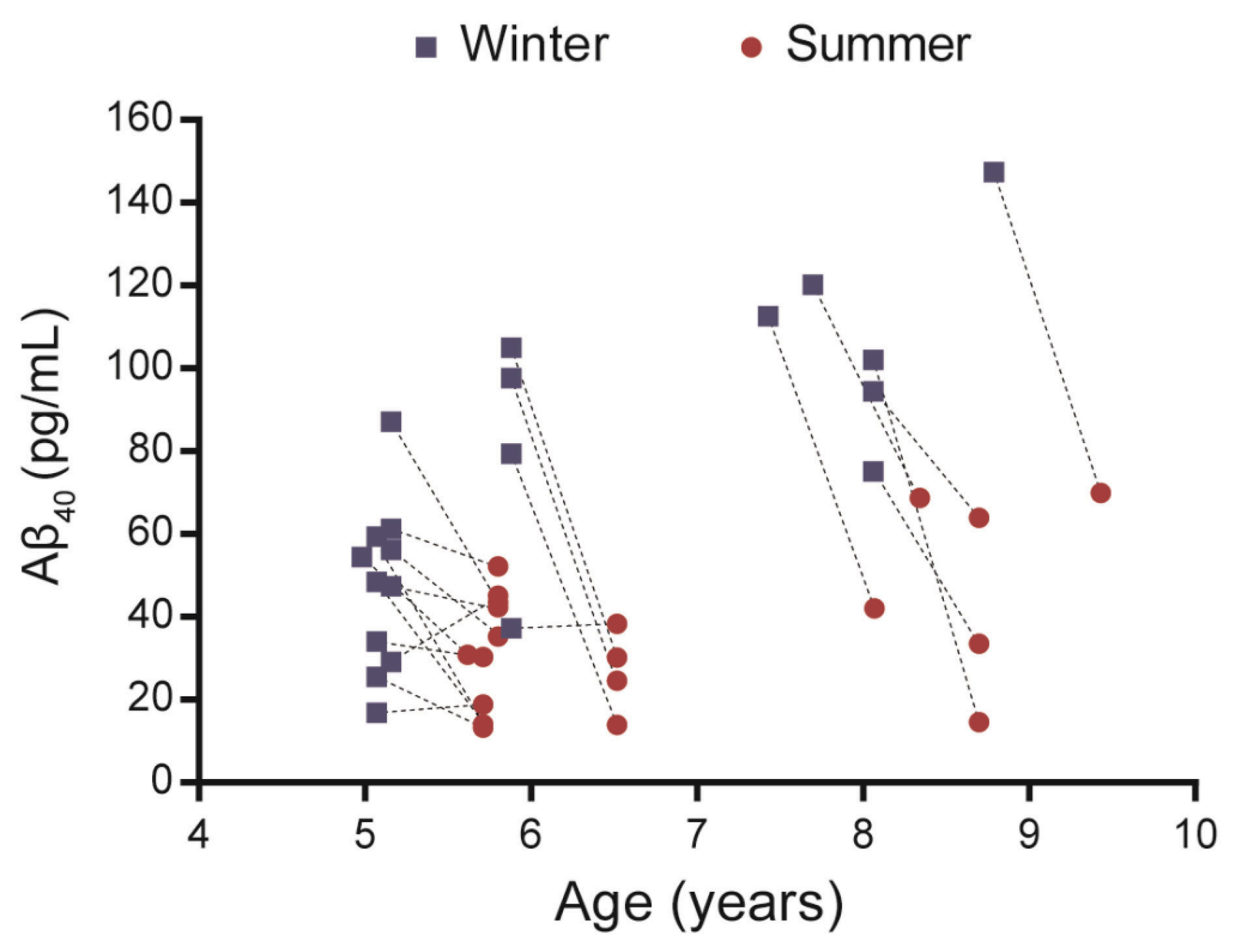
Evaluation of plasma Aβ_40_ levels in 21 mouse lemurs aged 5 to 9.5 years old. Aβ_40_ values were quantified in summer and winter (lines connect individual animal's results) and most animals displayed a reduction of Aβ_40_ levels between winter and summer.

Plasma Aβ_40_ was evaluated in 21 middle-aged or old mouse lemurs in winter and summer (Figure 1, Supplementary Table 1). Visual observation of the data revealed that, in most animals, Aβ_40_ levels decreased from the winter to the summer season.

**Figure 2:**
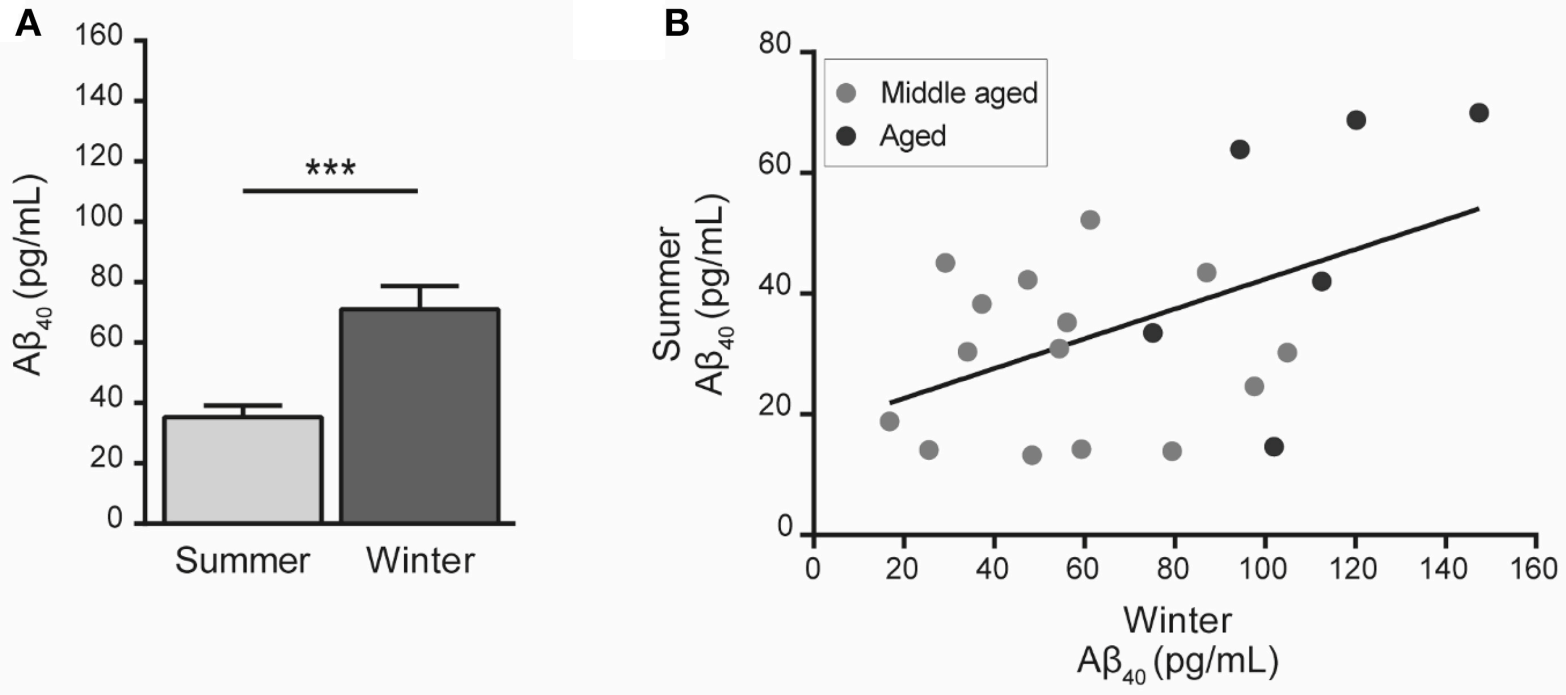
Plasma Aβ_40_ levels in mouse lemurs regardless of the age category. **(A)** Pooled data from middle-aged and old animals showed higher Aβ_40_ concentration in winter than in summer (paired Student's *t*-test *t* = 5.32, *df* = 20, *p* = 0.00003). ^***^p < 0.001. **(B)** Correlation between Aβ_40_ concentrations in summer and winter periods (Pearson's test, *R*^2^ = 0.23, *p* < 0.026).

**Figure 3:**
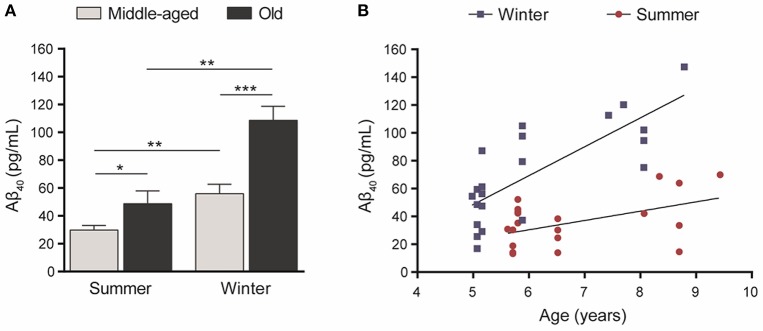
**(A)** Plasma Aβ_40_ levels in middle-aged (5–6 years old at the first sampling time, *n* = 15) or old (7.5–9.5 years old, *n* = 6) mouse lemurs. The plasma Aβ_40_ concentration was higher in old animals than in middle-aged ones in summer (*t* = 2.45, *df* = 19, *p* = 0.024) and winter (*t* = 4.19, *df* = 19, *p* = 0.0005). Also, the plasma Aβ_40_ concentration was higher in winter than in summer in middle-aged (*t* = 3.51, *df* = 14, *p* = 0.003) and old animals (*t* = 6.62, *df* = 5, *p* = 0.001). ^*^p < 0.05; ^**^p < 0.01; ^***^p < 0.001. **(B)** Age-associated evolution of plasma Aβ_40_ as a function of season. Plasma Aβ_40_ levels increased as a function of age during both seasons (Pearson's tests, winter: *R*^2^ = 0.60, *p* = 0.00004; summer: *R*^2^ = 0.24, *p* = 0.025).

Statistical analysis showed that Aβ_40_ concentrations in winter were higher than in summer regardless of the age of the animals (paired Student's *t*-test *t* = 5.32, *df* = 20, *p* = 0.00003, Figure 2A) and were also correlated between the two seasons (*R*^2^ = 0.23, *p* < 0.026, Figure 2B). When data were split into age categories, we found higher plasma Aβ_40_ concentrations in winter than in summer in both middle-aged and old animals (*p* = 0.003 and *p* = 0.001, respectively, Figure 3A).

Also, plasma Aβ_40_ was higher in the old compared to middle-aged animals and we found age-related increases of Aβ concentrations of 94% (*p* = 0.0005) and 64% (*p* = 0.024) in winter and summer, respectively (Figure 3A). Finally, plasma Aβ_40_ concentrations were correlated with age in both the winter (*R*^2^ = 0.60, *p* = 0.00004) and summer (*R*^2^ = 0.24, *p* = 0.025) periods (Figure 3B).

### Plasma Aβ_40_ seasonal variations are modulated by aging

**Figure 4:**
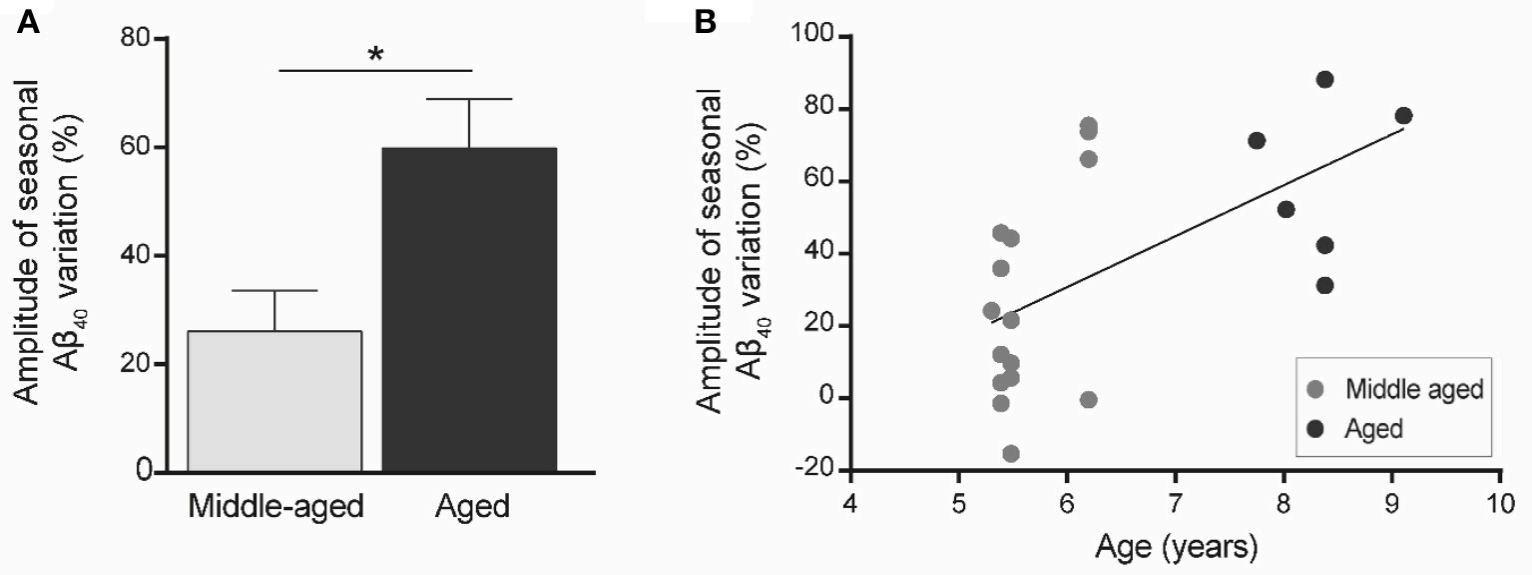
Amplitude of seasonal variations of plasma Aβ_40_ (ΔAβ_40_) in mouse lemurs (*n* = 21 animals). **(A)** Seasonal variations of plasma Aβ_40_ were greater in old compared to middle-aged animals. ^*^p < 0.05. **(B)** Seasonal variation of plasma Aβ_40_ is correlated with age in mouse lemurs (Pearson's test, *R*^2^ = 0.36, *p* = 0.004).

We then quantified the variation in plasma Aβ_40_ concentrations in the two seasons for each animal. Increased plasma Aβ_40_ seasonal variation (ΔAβ_40_) was found in old animals (Figure 4A) and ΔAβ_40_ was significantly correlated with aging (*R*^2^ = 0.36, *p* = 0.004, Figure 4B).

### Plasma Aβ_40_ and cerebral atrophy

**Figure 5:**
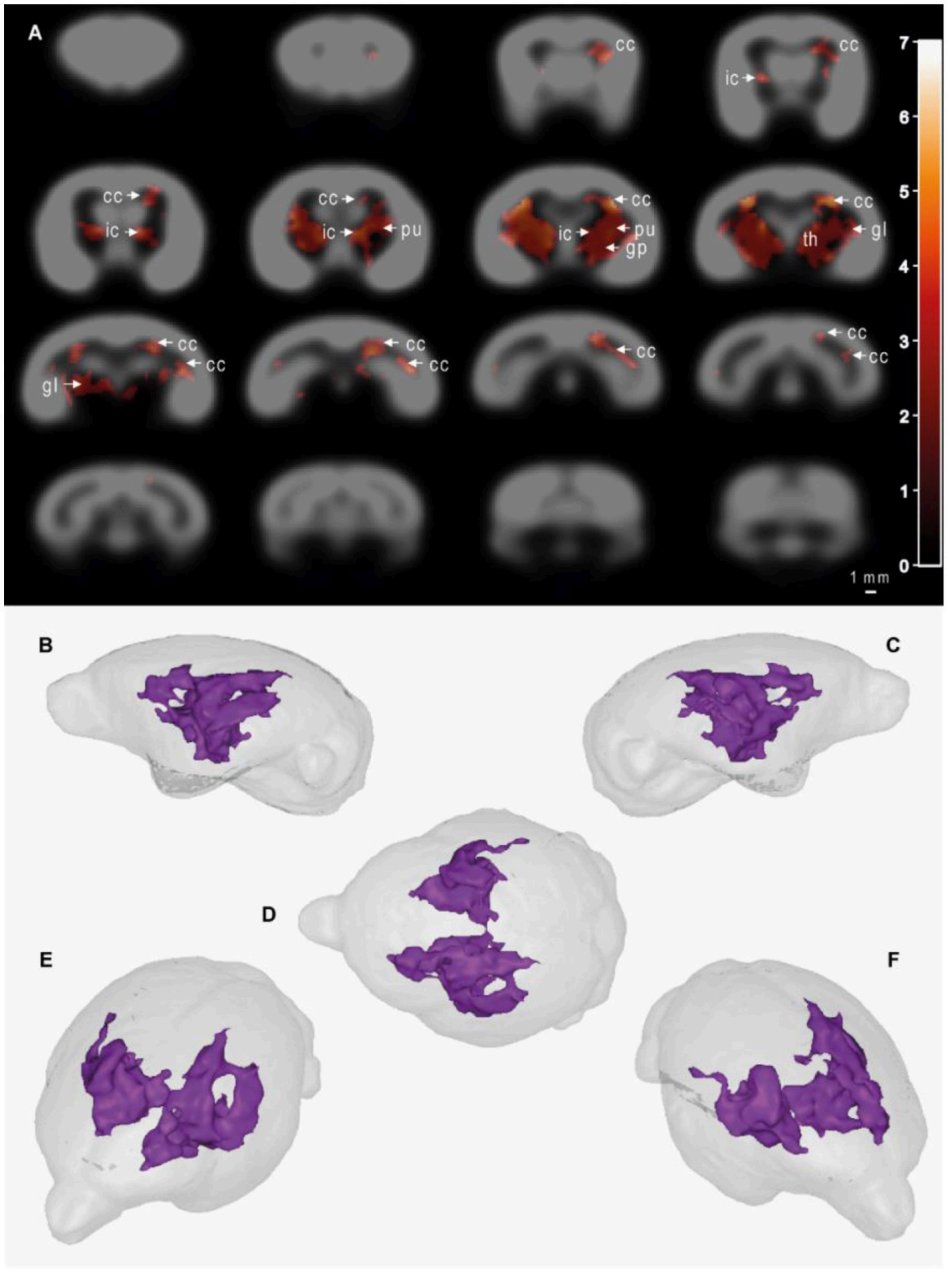
Cerebral alterations correlated with plasma Aβ_40_ levels in mouse lemurs. Statistical parametric maps depicting regions in which the probability of voxels belonging to white matter or subcortical nuclei increases with Aβ_40_ plasma levels **(A)**. In other words, the voxels in which low Aβ40 levels were significantly associated with atrophy of white matter and subcortical brain regions are shown as colored patterns on brain templates (i.e., gray scale images of the brain). Slices are spaced 1 mm apart along the rostro-caudal axis (VBM parameters: FDR-corrected *p* < 0.05; extent threshold *k* = 500; cluster mapping represents *t* values). Maps were derived from MRI recorded on 21 animals aged from 5 to 9.5 years old. The color bar represents the value of the *t* statistic (no unit). The cluster measured 104 mm^3^, so 8,516 pixels. cc, corpus callosum; ic, internal capsule; pu, putamen; gp, globus pallidus; th, thalamus; gl, geniculate nucleus. **(B–F)** 3D representations of affected areas: sagittal left **(B)**, sagittal right **(C)**, dorsal **(D)**, oblique front left **(E)** and oblique front right **(F)** views. Scale bar = 1 mm.

MRI images were used to evaluate, thanks to voxel-based analyses, the relationships between the age of the animals (GLM-1 including only an age effect and GLM-2 including age and plasma Aβ_40_ effects) or plasma Aβ_40_ levels (adjusted for the age of the animals - GLM-2) and cerebral atrophy [assessed from reduction in tissue probability maps (tpm)]. We did not detect any significant relationship between the age of the animals and gray matter, white matter or subcortical nuclei atrophy (analyses based on GLM-1 or GLM-2). We found a negative relationship between plasma Aβ_40_ levels and white matter and subcortical nuclei atrophy when both summer and winter Aβ_40_ were included in the model (GLM-2 model adjusted for the age of the animals, Figure 5) or when only summer Aβ_40_ was taken into account (Supplementary Figure 1). In other words, low Aβ_40_ levels were associated with atrophy of white matter and subcortical brain regions. This relationship involved a large cluster encompassing bilaterally the following brain regions: corpus callosum, internal capsule, putamen, globus pallidus, thalamus, and geniculate nucleus. No other relationships, i.e., negative relationships between plasma Aβ_40_ levels and white matter or subcortical nuclei tpm or any relationships between plasma Aβ_40_ levels and GM tpm were detected. Also, with GLM-2, we did not detect any age effect when plasma Aβ_40_ levels were used as variables of no interest.

## Discussion

This study evaluated plasma Aβ_40_ levels in a cohort of 5–9.5 year-old mouse lemurs as well as the relationship between plasma Aβ_40_ and cerebral atrophy. Mouse lemur primates express Aβ peptides homologous to those of humans (Silhol et al., 1996). However, amyloid plaque load is usually low in lemurs and only 10% of lemurs over 6 years old develop, mostly sparse, intracerebral Aβ deposits (Mestre-Frances et al., 2000; Kraska et al., 2011). In addition, the animals involved in this study were followed up by a veterinarian and they did not display any obvious pathology. Data from this study thus reflect “normal” aging conditions rather than pathological or AD-type conditions.

First, we found an age-related increase in plasma Aβ_40_ levels. Second, we found that seasonal rhythms have a major impact on plasma Aβ_40_ concentration with higher Aβ_40_ levels in winter, the season of low activity and torpor. We also showed that the difference between plasma Aβ_40_ levels in winter and summer (ΔAβ_40_) increases with age. Finally, evaluation of cerebral atrophy showed that aging was not associated with cerebral atrophy in the middle aged/old animals from this study. Interestingly, when data were adjusted for age, low Aβ_40_ levels were associated with white matter and subcortical nuclei atrophy. Thus, at a given age, animals with lower plasma Aβ_40_ levels were more atrophied than animals with higher Aβ_40_ values.

Our first result showing that old animals display higher plasma Aβ_40_ levels than middle-aged ones confirms a trend reported in a previous study in lemurs (Roy et al., 2015). It is consistent with data in humans, in which aging increases plasma Aβ (Lopez et al., 2008; Huang et al., 2013), although this result is not reported by all studies (Roher et al., 2009). It is also consistent with the age-related increased synthesis of Aβ_40_ reported in humans as well as primates (Fukumoto et al., 2004).

Since mouse lemurs are seasonal animals (Dal-Pan et al., 2011), it was important to determine the possible influence of seasonal variation on plasma Aβ_40_ levels. We found strong seasonal variations of plasma Aβ_40_ levels with increased concentrations during winter. To our knowledge, this is the first study reporting seasonal variations of Aβ in biological fluids. In humans, some studies (Huang et al., 2013), but not all (Lachno et al., 2009), showed diurnal variations of plasma Aβ, suggesting a circadian regulation of Aβ levels. Circadian variations of Aβ were also reported in mice, showing that interstitial fluid (ISF) Aβ levels are ~25% higher during the dark period, when rodents are more active, than during the light period (Kang et al., 2009). Fluctuations of plasma Aβ have been reported during a longitudinal study in humans (Roher et al., 2009), but their seasonal origin has not been established. Deciphering the biological parameters leading to the reported seasonal changes remains difficult. These changes could be associated with the regulation of the production and/or clearance of cerebral Aβ, as well as with regulation of the production and/or clearance of peripheral sources of Aβ.

Several hypotheses can be proposed to explain our results. We can rule out that an increased concentration of plasma Aβ_40_ in winter is linked to an increased production of Aβ. Indeed, in mouse lemurs, winter is associated with torpor and a reduction of general metabolism (Perret and Aujard, 2001; Dal-Pan et al., 2011), and thus with low neuronal activity. This hypoactivity should lead to a reduction of amyloid production as Aβ production is linked to neuronal activity (Cirrito et al., 2005). Also, we expect that the low general metabolism should lead to reduced Aβ synthesis from peripheral sources of Aβ such as the skeletal muscle, platelets or vascular walls (Roher et al., 2009). Another explanation for increased plasma Aβ_40_ in winter is increased clearance from the brain to the blood. In many animals, torpor leads to a reduction of the size of the neurons and of dendrite density and thus to increased ISF space (Magariños et al., 2006; von der Ohe et al., 2006; Andrews, 2007). Also, increased ISF space facilitates the clearance of various metabolites, including Aβ, to outside of the brain (Xie et al., 2013). Thus, one likely explanation for the increased plasma Aβ concentration in winter is a torpor-induced increase in ISF space leading to increased Aβ clearance in the blood. Reduced seasonal changes of peripheral Aβ clearance can also explain our results. As renal function was recently shown to participate in Aβ clearance (Jin et al., 2017), we cannot exclude that a lower renal function associated with the hypoactivity occurring in winter participated in the induction of high plasma Aβ levels. In humans, physical activity reduces plasma Aβ levels (Stillman et al., 2017), so the lower plasma Aβ level in summer, i.e., the active season for mouse lemurs, could be related to an increased physical activity. Follow-up studies on new cohorts of animals will have to be initiated to further evaluate the origin of seasonal changes in plasma Aβ_40_.

Then, we evaluated age-related changes of seasonal variation of plasma Aβ_40_ (i.e., the difference between plasma Aβ_40_ levels in winter and summer: ΔAβ_40_). ΔAβ_40_ increased with age. This result was not expected as the amplitude of most seasonal cycles (weight, metabolism, hormones, and so on) tends to decrease with aging in mouse lemurs (Perret and Aujard, 2006). Also, in humans, the amplitude of circadian plasma Aβ concentrations reduces with aging (Huang et al., 2013). In summer, old mouse lemurs already had plasma Aβ_40_ levels 64% higher than levels of middle-aged animals. In winter, this difference increased to 94%, leading to the increased ΔAβ_40_ in old lemurs. Because of the reduction of the general metabolism occurring in lemurs during winter, we do not expect any increased cerebral or peripheral Aβ synthesis in the old animals in winter. An additional explanation for the increased ΔAβ_40_ in old lemurs might be that in winter brain-to-blood Aβ clearance is enhanced and clearance from the blood is reduced. Mechanisms leading to these changes remain to be explored.

Our result showing that low Aβ_40_ levels are associated with atrophy in several white matter and subcortical brain regions was unexpected. This result was not linked to a confounding effect of aging as our analysis was adjusted for the age of the animals (taken as a variable of no interest). Aging did not modulate cerebral atrophy in our study either. Thus, the relationship between plasma Aβ_40_ and atrophy was independent of aging. However, our study suggests that plasma Aβ_40_ measures alone should not be taken as a surrogate marker for cerebral atrophy as it evolves with the age of the animals. In other words, plasma Aβ_40_ increases with aging and at a given age lower Aβ_40_ levels are associated with cerebral atrophy. In a previous study, we showed that low plasma Aβ_40_ levels in mouse lemurs are associated with high cerebral intracellular labeling with antibodies specific to amyloid and APP (Roy et al., 2015). Thus, a link between amyloid overproduction, low Aβ_40_ plasma levels and white matter and subcortical atrophy cannot be excluded. In humans, data evaluating the relationships between plasma Aβ and cerebral impairments are controversial. Several studies suggest a relationship between high plasma Aβ_40_ levels and cerebral pathologies such as white matter lesions (van Dijk et al., 2004; Gurol et al., 2006; Janelidze et al., 2016), hippocampal atrophy and increased rate of cerebral atrophy (Kaffashian et al., 2015). It is possible that these studies were enriched with AD patients and did not reflect non-pathological conditions. One study focusing on dementia-free older adults reported that low plasma Aβ_40_ levels were associated with increased progression of white matter hyperintensities (WMH) due to vascular alterations. The authors suggested that the association of low plasma Aβ_40_ levels with increased WMH may reflect increased deposition of Aβ_40_ in cerebral vessel walls after its clearance, resulting in impaired cerebral blood flow (Kaffashian et al., 2014). In our study we did not observe any obvious white matter hyperintensity, but the image resolution in mouse lemurs impedes the detection of such lesions. We cannot, however, rule out vascular alterations in our mouse lemurs. To date, our animals are involved in a longitudinal follow-up study, hindering access to tissue. To identify the cause of plasma Aβ_40_ lowering in the atrophied animals, new investigations are now required. Developing techniques for perfusion measurements combined with brain vessel histopathology should be an elegant way to link plasma Aβ and brain morphology variations and to determine the potential of plasma Aβ_40_ as a biomarker of brain pathology.

To conclude, we confirmed an age-related increase of plasma Aβ and showed for the first time that plasma Aβ_40_ is modulated by seasonal variations. Seasonal effects should thus be taken into account when studying plasma Aβ, in preclinical as well as in clinical studies. Since seasonal changes are amplified in mouse lemurs, they appear to be a useful model for evaluating the impact of seasons on amyloid metabolism. Further mechanistic studies should now be initiated to understand the origin of the link between plasma Aβ_40_ concentrations and cerebral atrophy as well as of the plasma Aβ changes associated with seasonal variations. We also observed that in normal conditions and after adjustment for age, low plasma Aβ_40_ is correlated with brain tissue atrophy in several white matter and subcortical regions including the corpus callosum, internal capsule, putamen, globus pallidus, thalamus and geniculate nucleus. The origin of these changes remains to be evaluated but supports a strong link between plasma Aβ_40_ concentrations and brain morphometry.

## Author contributions

CG and MD designed the study; CG and ZH performed the blood sampling; CG and A-SH performed the biochemical analysis; CG, A-SH, and MD were responsible for statistical analyses and wrote the manuscript; A-SH and MD performed the MRI voxel wise analysis; ZH revised the manuscript.

### Conflict of interest statement

The authors declare that the research was conducted in the absence of any commercial or financial relationships that could be construed as a potential conflict of interest.
