# Supplementary material for: Plasma Amyloid Is Associated with White Matter and Subcortical Alterations and Is Modulated by Age and Seasonal Rhythms in Mouse Lemur Primates
Source: Front Aging Neurosci. 2018 Feb 14;10:35. doi: 10.3389/fnagi.2018.00035 (PMC5817060; doi:10.3389/fnagi.2018.00035)
Supplement: Supplementary file 1 [file Image1.PDF]

Plasma amyloid is associated with white matter and subcortical alterations and is modulated by age and seasonal rhythms in mouse lemur primates

Charlotte Gary, Anne-Sophie Hérard, Zoé Hanss, Marc Dhenain

Supplementary Material

Supplementary Figure 1.

Cerebral alterations correlated with summer plasma $A\beta_{40}$ levels in mouse lemurs. Statistical parametric maps depicting regions in which the probability of voxels to belong to white matter or subcortical nuclei increases with summer $A\beta_{40}$ plasma levels (A). Slices are spaced 1 mm apart along the rostro-caudal axis (voxel-based morphometric parameters: FDR-corrected $p < 0.05$; extent threshold $k = 500$; cluster mapping represents T values). Maps were derived from MRI recorded on 21 animals aged from 5 to 9.5 years. The color bar represents the value of the t statistic (no unit). The cluster measured 93 mm^3, *i.e.* 7666 pixels. Abbreviations: cc: corpus callosum; ic: internal capsule; pu: putamen; gp: globus pallidus; th: thalamus; gl: geniculate nucleus. (B-F) 3D representations of affected areas: sagittal left (B), sagittal right (C), dorsal (D), oblique front left (E) and oblique front right (F) views. Scale bar = 1 mm.

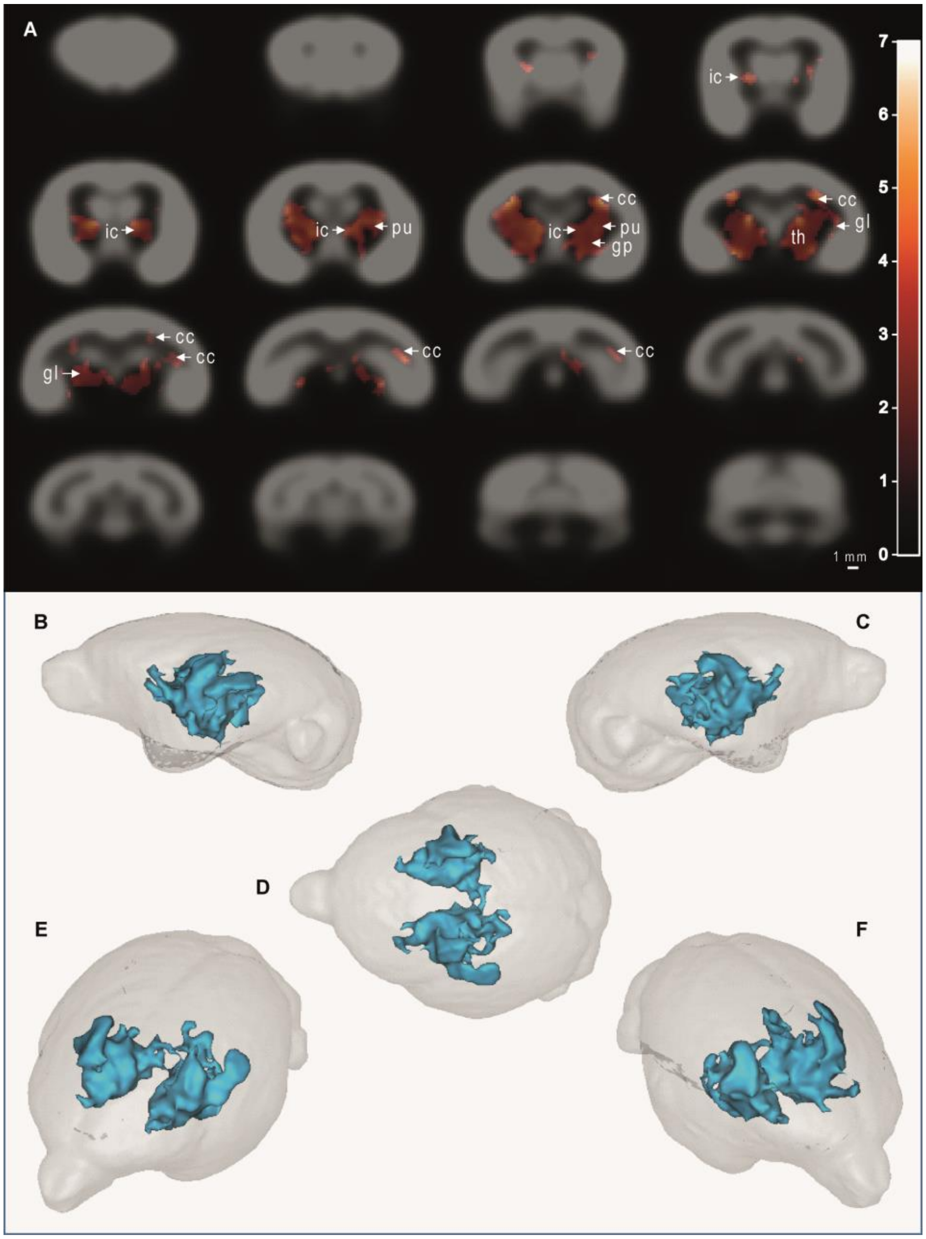
A
ic
ic
ic
pu
cc
ic
pu
gp
cc
th
gl
cc
cc
gl
cc
cc
7
6
5
4
3
2
1
0
1 mm
B
C
D
E
F
