# Supplementary material for: Plasma Amyloid Is Associated with White Matter and Subcortical Alterations and Is Modulated by Age and Seasonal Rhythms in Mouse Lemur Primates
Source: Front Aging Neurosci. 2018 Feb 14;10:35. doi: 10.3389/fnagi.2018.00035 (PMC5817060; doi:10.3389/fnagi.2018.00035)
Supplement: Supplementary file 2 [file Table1.PDF]

Plasma amyloid is associated with white matter and subcortical alterations and is modulated by age and seasonal rhythms in mouse lemur primates

Charlotte Gary, Anne-Sophie Hérard, Zoé Hanss, Marc Dhenain

Supplementary Material

Supplementary Table 1. Plasma Aβ_{40} concentrations in 21 middle-aged or old mouse lemurs measured in winter and summer. Presented data are the means of the duplicate.

Winter		Summer		Sex
**Age (years)**	**Aβ (pg/mL)**	**Age (years)**	**Aβ (pg/mL)**	
4.98	54.44	5.62	30.87	F
5.07	25.58	5.71	14.09	M
5.07	59.33	5.71	14.26	F
5.07	34.07	5.71	30.39	F
5.07	48.43	5.71	13.23	M
5.07	16.82	5.71	18.83	M
5.16	61.26	5.80	52.24	M
5.16	29.18	5.80	45.12	M
5.16	47.42	5.80	42.33	M
5.16	56.17	5.80	35.23	F
5.16	87.08	5.80	43.51	F
5.88	97.69	6.52	24.62	F
5.88	37.27	6.52	38.29	M
5.88	79.41	6.52	13.92	M
5.88	104.95	6.52	30.24	M
7.43	112.55	8.07	42.03	M
7.70	120.24	8.34	68.74	F
8.06	94.46	8.70	63.92	F
8.06	75.19	8.70	33.53	F
8.06	102.04	8.70	14.60	F
8.79	147.45	9.43	69.98	F
